# Automated inter-device 3D OCT image registration using deep learning and retinal layer segmentation

**DOI:** 10.1364/BOE.493047

**Published:** 2023-06-27

**Authors:** David Rivas-Villar, Alice R. Motschi, Michael Pircher, Christoph K. Hitzenberger, Markus Schranz, Philipp K. Roberts, Ursula Schmidt-Erfurth, Hrvoje Bogunović

**Affiliations:** 1 Centro de investigacion CITIC, Universidade da Coruña, 15071 A Coruña, Spain; 2 Grupo VARPA, Instituto de Investigacion Biomédica de A Coruña (INIBIC), Universidade da Coruña, 15006 A Coruña, Spain; 3 Medical University of Vienna, Center for Medical Physics and Biomedical Engineering, Vienna, Austria; 4 Medical University of Vienna, Department of Ophthalmology and Optometry, Vienna, Austria; 5 Medical University of Vienna, Department of Ophthalmology and Optometry, Christian Doppler Lab for Artificial Intelligence in Retina, Vienna, Austria; 6 david.rivas.villar@udc.es; 7 hrvoje.bogunovic@meduniwien.ac.at

## Abstract

Optical coherence tomography (OCT) is the most widely used imaging modality in ophthalmology. There are multiple variations of OCT imaging capable of producing complementary information. Thus, registering these complementary volumes is desirable in order to combine their information. In this work, we propose a novel automated pipeline to register OCT images produced by different devices. This pipeline is based on two steps: a multi-modal 2D en-face registration based on deep learning, and a Z-axis (axial axis) registration based on the retinal layer segmentation. We evaluate our method using data from a Heidelberg Spectralis and an experimental PS-OCT device. The empirical results demonstrated high-quality registrations, with mean errors of approximately 46 µm for the 2D registration and 9.59 µm for the Z-axis registration. These registrations may help in multiple clinical applications such as the validation of layer segmentations among others.

## 1. Introduction

Nowadays, OCT (Optical Coherence Tomography), is the gold standard imaging technique that allows to capture high-resolution cross-sectional images of the human retina in a non-invasive manner [1,2]. However, OCT methods still have some shortcomings. For instance, the lack of tissue-specific contrast complicates tissue differentiation [3]. This is aggravated in the presence of diseases that alter the retinal layers, causing them to be damaged, displaced, and disrupted [4].

Polarization-sensitive OCT (PS-OCT) is an extension of OCT that provides extra information by using the polarization information of the reflected light [3–7]. This allows PS-OCT to generate tissue-specific contrast due to the particular optical features of the different retinal layers. Moreover, PS-OCT allows to quantitatively measure a sample’s polarization properties [8] using, e.g., the degree of polarization uniformity (DOPU) or the birefringence. Therefore, PS-OCT can reveal important tissue information that cannot be obtained using conventional OCT imaging.

OCT devices from different manufacturers have different imaging characteristics, like different resolutions, de-noising algorithms, capture speeds, extra data (i.e., OCTA), available multi-modal imaging, etc. These characteristics are a result of different design trade-offs OCT device manufacturers had to take. Therefore, combining the images of different devices is often desirable to obtain the best aspect of each, or to fuse complementary information. This is specifically relevant for research devices, where novel features are tested without placing as much importance on conventional OCT image quality. An example of this are experimental PS-OCT devices, which may produce lower quality images than commercial devices. The commercial devices achieve this higher image quality by incorporating specific algorithms and approaches to improve their image quality and signal-to-noise-ratio (such as B-scan averaging). Particularly, for the case of PS-OCT, it would be beneficial to combine its polarization capabilities, such as the DOPU signal with the high-quality OCT imaging from a commercial device. The extra data provided by the PS-OCT could help support or verify layer annotations on standard OCTs. Moreover, it could even be used to train deep neural networks to predict the PS-OCT images from the corresponding higher quality commercial OCT data [9]. Commonly, to combine data from different devices, the OCT images have to be mutually registered.

Image registration is the process of aligning images taken from different viewpoints, different devices or from different time frames. Normally, in this process, a pair of images are aligned, one of which is considered the *fixed* image, which is used as a reference, and the other is the *moving* image, which is transformed to match the fixed image. Registration is a fundamental task in medical image processing pipelines. It is highly significant due to the broad implications it has for clinical practice [10,11]. Medical image registration allows for simultaneous analysis of multiple images which, in turn, allows clinicians to draw more informed conclusions [12]. Currently, image registration is used in medicine for longitudinal studies, disease monitoring and even in computer-assisted diagnosis and treatment. Longitudinal studies help to study and monitor changes in the structure of the eye due to, for instance, disease progression. For the purpose of comparing longitudinal data (i.e., a sequence of images from the same patient captured at different points in time), spatial correspondences need to be established between images through image registration [13]. Therefore, image registration is a key component of longitudinal studies [14], facilitating retinal change detection to monitor pathological progression. This, in turn, can improve disease activity assessment. Furthermore, image registration also allows for the fusion of images from different modalities, devices, or protocols, which also enhances the information seen by clinicians, facilitating diagnosis [11]. Additionally, automated registration is of particular importance for these computer-assisted methods [15,16] as they are infeasible to be performed manually as part of busy clinical workflows.

Typically, image registration approaches have been divided into Feature-Based Registration (FBR) and Intensity-Based Registration (IBR) [17]. More recently, a novel approach has appeared stemming from the deep learning methods, direct parameter regression (DPR). FBR methods are based on keypoints which are distinctive spatial locations that can be found in the images that make up each registration pair. These keypoints can be matched between images, and their relative locations used to infer the geometric transformation capable of aligning the two images. The main advantage of FBR methods is that the use of keypoints makes the process explainable and that these keypoints can be detected across imaging modalities, enabling multi-modal registration. On the other hand, IBR approaches optimize a similarity metric between the intensity values of the images. There are multiple similarity metrics that can be used for this, depending on the application field, image modalities, etc. Finally, novel deep learning methods aim to directly predict the transformation using parameter regression [18,19]. In these methods, a neural network learns to directly infer the parameters of a transformation matrix or of a deformable transformation field. These approaches are trained using both the fixed and the moving images. The loss function is computed by transforming the moving image using the parameters predicted by the network and computing the similarity of this predicted image versus the expected one. Therefore, in order to minimize this loss function, the neural network learns to estimate the registration transformation parameters accurately.

Overall, OCT imaging lacks the variety and number of automated registration methods seen on other medical imaging modalities like Magnetic Resonance Imaging (MRI) or Computed Tomography (CT) [20]. This is mainly due to multiple OCT-specific image properties that complicate the registration process. For instance, OCT images are particularly affected by speckles [21–23] which can obscure retinal features [20]. Therefore, IBR methods are unsuitable and result in poor performance [20]. Similarly, lesions caused by disease can alter the aspect of the retina, which hampers the keypoint localization in FBR methods. Moreover, OCT images have higher spatial resolution than other medical imaging modalities (e.g., MRI), which increases the computational cost of registration [24]. All of these characteristics make OCT image registration a challenging task and complicate the adaptation of image registration techniques developed for other modalities, which are therefore unreliably on OCT volumes [20].

In this paper, we propose a novel 3D registration pipeline which can be applied to OCT images captured with different devices, which, to the best of our knowledge, is an issue that remains unaddressed by current research in the field. In particular, we empirically evaluate this pipeline using images from a Heidelberg Spectralis OCT and an experimental PS-OCT device from MedUni Wien. First, we propose an unsupervised deep learning multi-modal feature-based registration of the 2D en-face projection obtained from the PS-OCT intensity volume and the SLO image obtained in conjunction with the Spectralis OCT. The transformation that aligns these 2D images also aligns the full 3D volumes *en-face*. A second registration step allows to register each B-scan axially, completing the full 3D registration. Therefore, this pipeline allows the complementary information from different OCT devices to be fused, improving the quality of information available to clinicians. Our approach demonstrates accurate results and fast execution times.

## 2. Related work

Currently, in the field of natural image registration, the best performing approaches are deep learning-based FBR methods [25,26]. On the other hand, in medical image registration, IBR methods offered the best results classically [27]. Currently, novel DPR methods have started to produce state-of-the-art performance on many fields like MRI [19]. Retinal image registration is a field currently dominated by classical, non-deep learning methods [24,28–30]. However, there have been important recent advances in FBR-based deep learning methods. For instance, in color fundus registration, deep learning-based domain specific [31] or generic keypoint [32] registration approaches are able to obtain results comparable to the classical methods.

Due to the 3D nature of OCT volumes, the registration methods may have different scopes and goals. Most of the automatic proposals intend to register A-Scans or B-Scans in order to correct for eye movements [33–35]. Other approaches register several OCT en-face projections to obtain large field of view images [30]. In order to achieve a complete image-to-image 3D OCT registration, there are also several approaches. Chen et al. [36] proposed a two-step deformable registration method. Considering the geometry of the retina, they used a global rigid registration and assumed that the deformable transformation could only occur along A-scans. As this method is intensity-based it requires multiple pre-processing steps like intensity normalization, retinal boundary detection, fovea localization and masking. Similarly, Gong et al. [37] used the spatially region-weighted correlation ratio (SRWCR) as an alternative intensity-based metric for the registration. Since these methods are IBR, they have several shortcomings. They are sensitive to speckle noise and their computation complexity is usually very high, normally in the order of hours [24,36].

There are also FBR methods like the one developed by Bogunović et al. [38]. To increase the field of view with mosaicing, they used a 2D *en-face* alignment using SURF descriptors over the simultaneously acquired SLO images, as the OCT en-face projections have low resolution. However, such an approach is only possible for Spectralis OCT registration, where the SLO and the corresponding OCT are registered as part of the image acquisition process.

Finally, there are also multiple hybrid methods which combine IBR and FBR methods. Pan et al. [24] used layer segmentations instead of discrete keypoints, combining them with specific intensity and vessel features. To register the images, they first process the OCT images by flattening the retina, a process which removes the curvature of the retina but cannot be reversed [24]. Out of the total 11 retinal surfaces, they detect and use 7 of them, giving each a different importance coefficient, with the top and bottom surfaces (ILM, Internal Limiting Membrane, and the outer bound of the RPE, Retinal Pigment Epithelium) being the most important. Moreover, they also use *ad-hoc* intensity-based region features and, for each A-scan in the OCT, they estimate a measure of vesselness, obtained from 2D projections of the 3D OCT. These features are weighted and used in a hierarchical deformation to align the 3D OCT images. Due to the deformable nature of this method, smoothing techniques need to be used in order to reduce the discontinuities in the computed deformation. On the other hand, Wei et al. [39] first detect vessel keypoints in the 2D *en-face* projection. However, due to the method used to detect these keypoints, they can be confused with the effects of choroidal neovascularization, a retinal disease. Therefore, if this disease is present in the projection images, randomly selected intensities from the object boundary are used to fill the affected area. After this first pre-processing step, in order to detect the keypoints, another pre-processing step is done to enhance the vessels. Then, using classical morphology, the vessels are skeletonized. Using back-projection, these points can be located in the 3D space of the OCT image. Then these points are registered in 3D using the coherent point drift algorithm. Finally, this coarse registration is refined using an IBR approach based on normalized mutual information.

It should be noted that none of the methods that have been previously described were specifically designed or validated for the purpose of registering OCT images that were acquired using different OCT devices. Therefore, our proposed method represents the first attempt in the field to address this task.

In multi-modal retinal image registration, current methods are generally feature based in order to overcome the differences among the imaging modalities. Commonly these approaches rely on classical keypoint detectors and descriptors such as different variations of SIFT [40–42], SURF [43,44], HOG [43,45,46], LoSPA [18] or PIIFD [40,44]. Similarly, other works use domain-specific keypoints like Hervella et al. [47] who use blood vessel crossovers and bifurcations, or Golkar et al. [48] who use the border of major blood vessels. Furthermore, both of these works have a subsequent intensity-based deformable registration step to refine the results. This extra step can refine the registrations, although it cannot improve the cases where the original method failed [47]. Classical approaches commonly require pre-processing so that keypoints and their descriptors can properly match among the different modalities.

More recently, several works [49–52] have tested deep learning approaches. Some methods leverage the capabilities of deep learning to improve the classical pipelines. For instance, Lee et al. [50] proposed a deep learning approach which uses Step Pattern Analysis learned via a CNN. They create patches centered on vessel intersection points, and, during the inference, the keypoints are described according to the learned patterns. On the other hand, other methods propose a full deep leaning pipeline like De Silva et al. [49] who use a parameter regression network, based on the comparison of features previously extracted by a separate Siamese network. Using these features, the network predicts affine deformations in small multi-modal images ( 200×200 ).

Sindel et al. [51] used a two-headed network capable of detecting keypoints and creating their cross-modal descriptors. This network is joined with SuperGlue [53], a graph-neural network capable of point matching, to create an end-to-end training with losses dedicated to the keypoints, the descriptors and their matching. An et al. [52] proposed to refine SuperPoint [26], a state-of-the-art keypoint detector and descriptor network in natural images. To achieve this, they use the vessel segmentation of multi-modal images. After the keypoints are detected and described, an outlier-rejection network is used to prune them prior to the transformation estimation.

In summary, deep learning image registration approaches offer improvements over the classical ones. Namely, end-to-end training without parameter tuning, as well as more flexibility and robustness to different imaging modalities. In addition, they can adjust to other challenging conditions, e.g., the evolution of pathological lesions which alters the retinal appearance. These properties make deep learning preferable over classical methods. For that purpose, we created a two-step 3D OCT registration approach based on deep learning. Our approach is the first deep learning method for OCT *en-face* registration. We propose to use a joint detector and descriptor network for the multi-modal registration of the SLO and *en-face* projection images from different OCT scans. The full 3D registration is then completed using the retinal layer segmentations of the OCT volumes as reference, adjusting the volumes A-scan-wise (Z-axis).

## 3. Methods

**Fig. 1.**
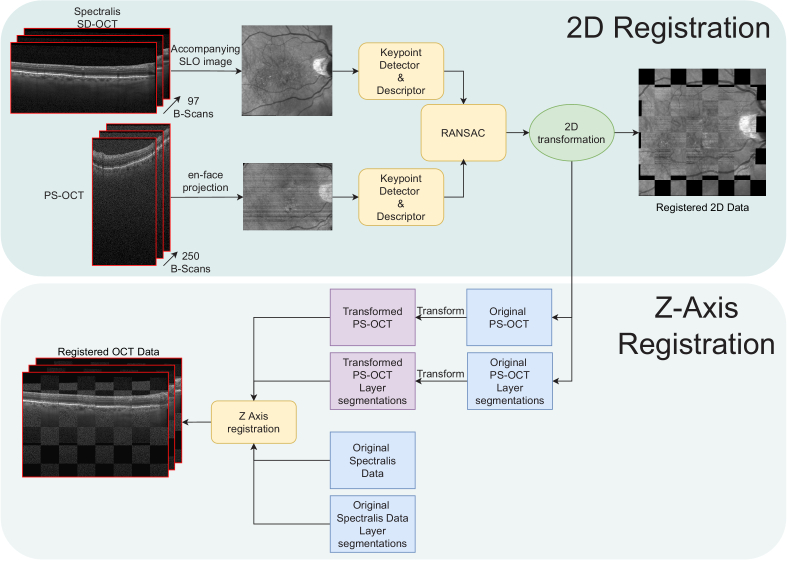
Overview of the proposed two-step 3D registration methodology. The top-most part is the 2D registration step, based on a joint detection and description neural network. The bottom part corresponds to the layer-based Z-axis registration.

A complete overview of the proposed two-step registration pipeline can be seen in Fig. 1. In brief, the pipeline starts from two 3D OCT volumes, acquired at the same visit but by two different devices. The first step consists of a 2D registration, which uses a 2D representation of the original volumes, e.g., an *en-face* OCT projection or a corresponding and aligned SLO image. We propose to use an unsupervised deep learning method based on a current state-of-the-art approach, previously shown to be successful both for natural [25] and retinal images [32]. This method is capable of both detecting and describing keypoints at the same time. After matching their descriptors, the paired keypoints are used to estimate the transformation using RANSAC [54]. Once the 2D registration is complete, the two 3D volumes are considered registered *en-face*, which means that they aligned in two of the three necessary axes. Therefore, to fully register the volumes, we propose a method which uses the ILM layer segmentation in order to align each B-scan with its correspondence. This allows us to exactly map the data from one OCT volume to another and thus complete the 3D OCT registration.

In particular, for the development and experimental evaluation of this method, we used OCT volumes captured by different devices, a commercially available Heidelberg Spectralis and a custom-built PS-OCT. The Spectralis scans include an SLO image, which helps mitigate the low spatial resolution of the *en-face* OCT projection produced by this device. Therefore, the proposed 2D registration matches the SLO from the Spectralis OCT and the *en-face* OCT projection from the PS-OCT device.

### 3.1 Deep learning-based 2D registration

For the first step, i.e., the 2D registration, we propose to use R2D2 [25]. R2D2 is capable of jointly detecting and describing keypoints using a single fully convolutional neural network. This network produces three different outputs. First, X , which is a dense set of descriptors composed of 128 channels. These descriptors correspond one-to-one with each pixel in the original en-face input image. Second, R is a reliability heatmap whose function is to estimate how discriminative the descriptor of each pixel is. Finally, the third output is a repeatability heatmap ( S ) which acts as the base keypoint detector. To select the keypoints using this map, a local maxima is employed, thus selecting only the most salient points. It should be noted that this heatmap is trained to produce maximal points that can be detected among both images in a registration pair, as detailed in the section 3.1.1. The keypoints are processed using the reliability heatmap, discarding those with low reliability scores.

R2D2 uses a modified L2-Net as the CNN architecture [55]. The modifications are made by the original authors so that the output is of the same size as the input image [25]. We train the network using unlabeled images and simulated geometric transformations. In particular, for each training image, we generate an image pair $\left(I,I^{\prime }\right)$ , where I represents the original image and $I^{\prime }$ is the synthetically transformed image. From the transformations, an optical flow U is constructed. This flow indicates the correspondence between the points of each image in the pair. Both images I and $I^{\prime }$ are processed by the network, generating their descriptors ( X and $X^{\prime }$ ), reliability ( R and $R^{\prime }$ ) and repeatability ( S and $S^{\prime }$ ) heatmaps, respectively.

#### 3.1.1 Neural network training objectives or loss-terms

As in R2D2 [25], we train the network using two loss terms, one for the repeatability heatmap and the other for the reliability heatmap. The reliability loss is composed of the specific reliability term in combination with the AP loss, which is used to learn descriptors. Thus, the global loss is defined as:

(1) $$L=L_{rep}+L_{AP,R},$$

where $L_{rep}$ is the repeatability loss and $L_{AP,R}$ is the reliability loss.

The repeatability loss ( $L_{rep}$ ) incentivizes the creation of peaks or local maxima, while also incentivizing that said maxima are present on the reliability maps for both images in a registration pair. To do so, this loss is composed of a term to maximize the cosine similarity among the corresponding image heatmaps and a peakiness term to create keypoints on those heatmaps. Both losses are computed locally, in windows of size N×N obtained from both heatmaps S and $S^{\prime }$ . The peakiness term is calculated separately for the two inputs and then both values are averaged, fusing the loss from both inputs into a single value. Due to its per window computation, the peakiness loss effectively enforces the creation of a single peak in each window. Therefore, the window size controls the frequency of the detected keypoints.

Overall, the repeatability loss is the following:

(2) $$L_{rep}(S,S\prime ,U)=L_{cosim}(S,S\prime ,U)+\frac{1}{2}(L_{peaky}(S)+L_{peaky}(S\prime )),$$

where $L_{cosim}$ is the cosine similarity loss defined in Eq. (3) and $L_{peaky}$ is the peakiness loss defined in Eq. (4).

(3) $$L_{cosim}(S,S\prime ,U)=1-\frac{1}{|W|}\sum_{w\in W}cosim(S_{f}[w],S_{fU}^{\prime }[w]),$$

where $S_{f}[w]$ represents the flattened version of the N×N window w extracted from the heatmap S , and $S_{fU}^{\prime }[w]$ the flattened version of the corresponding window obtained from the transformed image S′ using U which represents the optical flow linking each point to their correspondences. cosim represents the cosine similarity.

(4) $$L_{peaky}(S)=1-\frac{1}{|W|}\sum_{w\in W}(\max S[w]-\text{mean}\;S[w]),$$

where W denotes the set of all possible overlapping windows of size N×N , S[w] a particular window w extracted from S , max computes the maximum value of a set, and mean the average.

On the other hand, the reliability loss, $L_{AP,R}$ , has two objectives. The first one is to optimize the descriptors such that the distance among descriptors of the same point is minimal and the distance between descriptors of distinct keypoints is maximal. The second one is to train the reliability heatmap R so that it can predict which points provide reliable descriptors that can provide an accurate matching.

As in R2D2, the descriptors are trained using the AP loss [25,56], based on a differentiable approach of the Average Precision (AP). In particular, for any pixel (i,j) in the original image I we can consider that the descriptor $X_{i,j}$ corresponds to the descriptor of a patch $p_{i,j}$ centered on that pixel, with a size of M×M , equivalent to the network’s receptive field. After the descriptor computation, the descriptor $X_{i,j}$ is compared with the descriptors from the transformed image, X′ . Then, taking into consideration the optical flow U , the AP is computed by assessing how well the true corresponding descriptor $X_{U,i,j}^{\prime }$ has been ranked in comparison with the other ones. Thus, this loss maximizes the AP between the descriptor and its true correspondence, forcing the distance between $X_{i,j}$ and $X_{U,i,j}^{\prime }$ to be less than to any of the other points in X′ .

(5) $$L_{AP}=\sum_{i,j}1-AP(p_{i,j})$$

where $p_{i,j}$ denotes a particular patch for which the AP is computed.

To train the reliability heatmaps an extra term is added to this AP loss creating the complete reliability loss:

(6) $$L_{AP,R}=\sum_{i,j}1-AP(p_{i,j})R_{i,j}-k(1-R_{i,j})$$

where k∈[0,1] is the AP threshold above which a descriptor is considered reliable. In that regard, when a patch is reliable (i.e., AP>k ) the loss incentivizes the maximization of the reliability ( R ). On the contrary, when a patch is unreliable (i.e., AP<k ) the loss minimizes the reliability. It should be noted that the reliability R has a unique value per pixel, therefore it can be defined as $R_{i,j}$ , where i and j are the indexes of any particular pixel. As the descriptors are also pixel-wise, this means that each descriptor has its own reliability value. The middle value of k=0.5 was found to be adequate in [25] and [32] and hence it is used in our work as well.

#### 3.1.2 Neural network inference and keypoint matching

After training, the network is used to obtain the keypoints and descriptors for multi-modal pairs of images. For this, we follow the method described in R2D2 [25,32]. First, for each image, an initial set of keypoints is selected automatically using a local maximum filter over the repeatability heatmap. Following the R2D2 method [25,32], we employ a multi-scale approach, computing the keypoints at different image scales. Therefore, for each image, the network is run multiple times, reducing the size of the image each time. This produces an initial set of keypoints which is subsequently refined by eliminating those with a low score in the reliability heatmap ( $R_{i,j}$ ).

After obtaining the keypoints, we match their descriptors using the Euclidean distance. The paired keypoints are used as input data for RANSAC. The number of inliers needed to create a transformation model depends on its complexity [57]. We propose to use an affine transformation, therefore, our model requires at least 3 inlier keypoints. The affine transformation provides a good compromise between the more restricted transformations (i.e., similarity) and the more complex ones, like projective or non-rigid ones, and has commonly been used in the state of the art [46,49,50].

### 3.2 Layer segmentation-based Z-axis registration

**Fig. 2.**
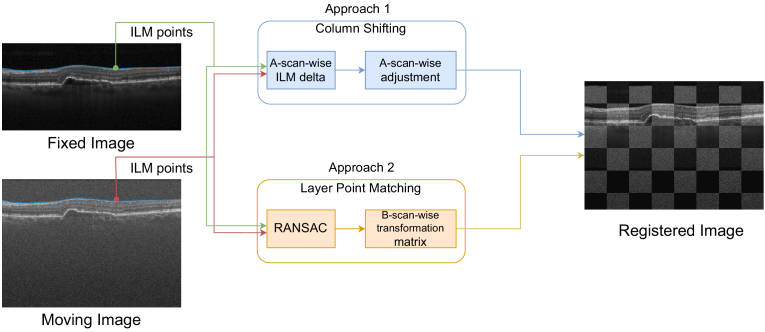
Schematic overview of the two proposed methods for the layer-based Z-axis registration.

After obtaining the 2D transformation, it can be used to align the full OCT volumes en-face. Given that the 2D registration was successful, the difference for each pair of corresponding OCT scans should be along their Z axis only. Therefore, to complete the volume alignment, each moving B-scan needs to be adjusted vertically to match the fixed one. To achieve this registration, we propose to employ the available retinal layer segmentations. In particular, we propose to use only the ILM as reference for the registration. The ILM is the top-most retinal layer and due to this positioning and large gradient, it is among the easiest retinal layers to detect and the most reliable one. Moreover, it has already been used in the state of the art as one of the main guiding layers (along with the outer bound of the RPE) in the method proposed by Pan et al. [24]. Using the ILM as reference, we propose two separate approaches, Column Shifting and Layer Point Matching (LPM). A schematic overview of both alternative approaches can be seen in Fig. 2.

Column Shifting is based on a column-wise deformation of the B-scans. It should be noted that each of the columns of a B-scan represents a unique A-scan, therefore, this deformation is also A-scan-wise. The proposed transformation is computed using the points belonging to the ILM as per the layer segmentation. The difference ( Δ ) between the ILMs of the fixed and moving B-scans is used to vertically and independently move each column. As the OCT volumes are registered in 2 out of 3 axes, this transformation would completely align the volumes. This approach can be mathematically formulated as:

(7) $$\Delta_{i,j}=ILM_{i,j}-ILM_{i,j}^{\prime T_{2D}},\forall j\in A,\forall i\in B,$$

where B is the set of B-scans in an OCT volume and A is the set of A-scans in each of these B-scans (i.e. columns). $ILM_{i,j}$ represents the points belonging to ILM of a particular B-scan ( i ) and A-scan ( j ) from the fixed image I . Likewise $ILM_{i,j}^{\prime T_{2D}}$ represents the points belonging to the ILM of a particular B-scan ( i ) and A-scan ( j ) of the moving image I′ with the 2D transformation ( $T_{2D}$ ) applied, $I^{\prime T_{2D}}$ . Therefore, the registered OCT volume can be computed simply by shifting each A-scan using the previously calculated $\Delta_{i,j}$ :

(8) $$I_{R}^{\prime }=I_{i,j}^{\prime T_{2D}}-\Delta_{i,j},\forall j\in A,\forall i\in B$$

where $I_{R}^{\prime }$ is the registered moving image ( I′ ), i.e., the registered OCT volume.

This method can perfectly match the ILM location in both B-scans, even compensating for acquisition errors. However, this proposal is sensitive to problems in the ILM segmentation, as it fits each column separately. Therefore, if a single pixel of the ILM segmentation is erroneous, the registration for that A-scan column will be wrong as well.

On the other hand, Layer Point Matching uses the points of the ILM as regular keypoints and matches them using RANSAC. As the images are registered in two of the three possible axes, the ILM points are already paired which should allow them to be directly used with RANSAC to easily infer the transformation that aligns both B-scans. This means that, in this step, we match the keypoints directly, without the descriptor computation. This approach can be formulated mathematically as:

(9) $$\tau =R(ILM_{i},ILM_{i}^{\prime T_{2D}},T),\forall i\in B,$$

where R represents the RANSAC algorithm. $ILM_{i}$ represents the points belonging to ILM of a particular B-scan i from the fixed image I . Likewise $ILM_{i}^{\prime T_{2D}}$ represents the points belonging to the ILM of a particular B-scan i of the moving image I′ with the 2D transformation ( $T_{2D}$ ) applied, i.e., $I^{\prime T_{2D}}$ . T is the transformation model to which RANSAC ( R ) fits the keypoints. Finally, τ is the set of resulting transformation matrices, one per B-scan.

Since the expected transformations among paired B-scans are small, we propose to test transformation models with few degrees of freedom, like rigid or similarity [57]. Therefore, this approach is resistant to errors in the ILM segmentation as it creates many possible keypoints, and can ignore the noisy ones.

## 4. Experiments

### 4.1 Dataset

We used the dataset from a longitudinal prospective clinical study (clinicaltrials.gov: NCT03838679) of 56 treatment-naïve patients with neovascular AMD, conducted at the Department of Ophthalmology and Optometry and the Center for Medical Physics and Biomedical Engineering (CMPBE) at the Medical University of Vienna [58,59]. This study was approved by the local ethics committee and adhered to the tenets of the Declaration of Helsinki. Every patient gave written informed consent prior to study inclusion, and only one eye from each patient was selected for imaging.

This dataset contains OCT scans from both a custom-built PS-OCT device [60] (Center for Medical Physics and Biomedical Engineering, MedUni Wien, Austria) and a commercial Spectralis OCT device (Heidelberg Engineering, Germany) acquired on the same visit day. All acquisitions were centered on the macula and contained B-scans with 1024 A-scans. The PS-OCT intensity and Spectralis volumes consisted of 250 B-scans and 97 B-scans, respectively, and covered an en-face area of 8×6 ${\mathrm{mm}}^{2}$ and 6×6 ${\mathrm{mm}}^{2}$ , respectively.

This data contains a one-year follow-up, where each patient has one or more visits. In each visit there might be PS-OCT data, Spectralis data, or both. From the patients enrolled, 52 of them had visits where both types of OCT images were acquired. Overall, in this subset, we could identify 119 pairs of OCT images. Therefore, to train the 2D registration model, we randomly split this data patient-wise 60-40% for the training and test sets, respectively. It should be noted that we do not use a validation set, as both steps of the method were trained using just the training set and their performance is evaluated on the test set. This results in a total of 88 visits from 33 patients for training, and 31 visits from 19 patients for testing.

### 4.2 Experimental details

For the first step, the 2D registration, we use the PS-OCT *en-face* projection and the SLO image which accompanies the Spectralis OCT. Using the SLO image has several advantages over using the OCT *en-face* projection, namely higher image quality and larger Field of View (FoV). As the Spectralis has fewer slices (97) in comparison to the PS-OCT (250), the resulting projection is of lower quality than the PS-OCT one. Moreover, the higher FoV captures more retinal features, which makes registration easier. However, the disadvantage of using the SLO is that it casts the 2D registration as a multi-modal problem, which is more complex.

Creating an *en-face* image from the PS-OCT intensity data consists in a dimensionality reduction from the original 3D volume to a 2D image. The quality of the *en-face* projection image determines the quality of the subsequent registration, as noise, shadows, etc. can negatively impact any registration method. Therefore, leveraging the great amount of information available within each PS-OCT intensity image, we propose to produce different projections and study their effect on the multi-modal 2D image registration.

In order to create high quality OCT projections, the layers of the retina are commonly used to define the boundaries of the regions to obtain the data from. Therefore, we use The Iowa Reference Algorithms (Retinal Image Analysis Lab, Iowa Institute for Biomedical Imaging, Iowa City, IA) [61–63], to create the layer segmentations for both the Spectralis data as well as the PS-OCT. Those algorithms are public, capable of segmenting eleven retinal surfaces, and are robust to image domain shift unlike many deep learning methods. It should be noted that these segmentations are also the ones used as reference in the second step, Z-axis registration.

Based on the PS-OCT intensity and layer data, we created four different projections:

1. **Outer projection**: To create this projection, we obtain the values between the locations of the ILM and outer bound of the RPE+20% of the length of the Z-axis. This translates to approximately 400 pixels below the location of the RPE. This addition allows for reduced noise and improved contrast in the projection. To collapse the 3D values of this area of the OCT scan into the 2D projection, we average them.
2. **Inner projection**: To compute this projection, we obtain all the values between 30 µm below the top of the Z axis and the RPE and average them.
3. **Hybrid projection**: In this projection, we propose to combine the upper bound of the inner projection (i.e., 30 µm below the top of the axial axis) and the lower bound of the outer projection (RPE+20% of the length of the Z-axis) to test if a combination of the features of the above projections is beneficial.
4. **Layer-based projection**: This projection gathers data between the Inner Segment/Outer Segment Junction (IS/OSJ) and the outer bound of the RPE. To compute the projection, these values are averaged.

**Fig. 3.**
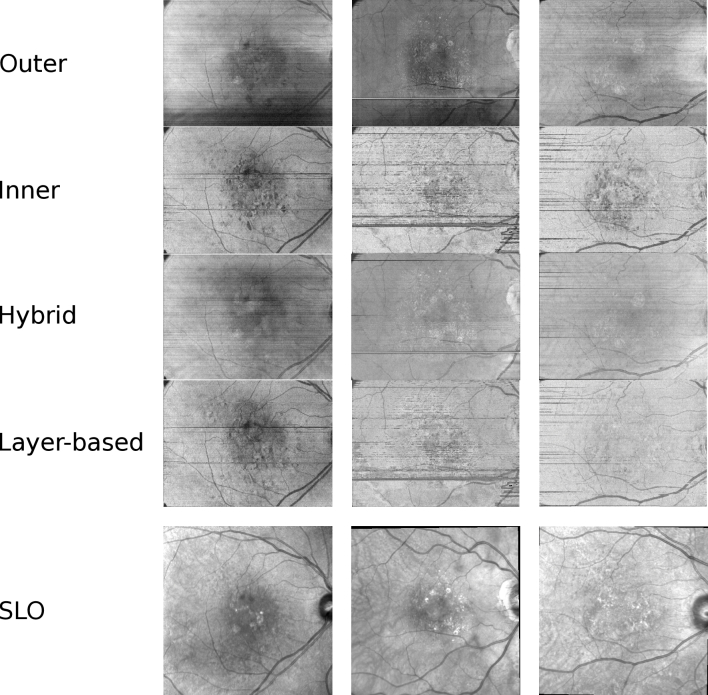
Examples of the different en-face projections for three patients (columns).

A visual example of the different projections in conjunction with their respective SLO images is shown in Fig. 3. As it can be observed in this figure, the different projections present a trade-off between contrast (highlighted arterio-venous tree against background) and clarity (reduced noise). Generally, the inner and the layer-based projections offer high contrast at the cost of increased punctual noise. On the other hand, the outer and the hybrid ones produce images that have less contrast and less noise. This noise, including points (salt and pepper noise), spots (zones larger than single points with low or null contrast) and horizontal streaks may confuse the keypoint detector and descriptor due to their prevalence, and therefore, can reduce the registration performance.

In terms of the training setup, we used the one found the best in a prior work that used R2D2 with retinal fundus images [32]. Particularly, the chosen optimization algorithm is Adam [64] with decay rates of $\beta_{1}=0.9$ and $\beta_{2}=0.999$ , the default values proposed by its authors. The learning rate is set to 0.0001, the weight decay to 0.0005 and a batch size of 8. We employ a fixed training regime of 2500 epochs to ensure stable convergence. We use a value of k=0.5 and N=16 as used in the state of the art [25,32]. Furthermore, we also keep the augmentation setting used in [32], randomly scaling the input image between 768 pixels and 256 and adding pixel noise and tilting for the in-pair augmentations. At inference the keypoints and descriptors are obtained as previously mentioned, running the network multiple times and within the same scales the network was trained in. It should be noted that, to compute the transformations, the keypoints are scaled to their original resolution, which is different in the cases of SLO images and PS-OCT intensity projections.

For the second step of the pipeline, Z axis registration, we tested two separate methodologies: column shifting and layer point matching. Within the layer point matching, we tested rigid and similarity transformations in order to find the most appropriate one for B-scan alignment.

### 4.3 Evaluation metrics

#### 4.3.1 2D registration

**Fig. 4.**
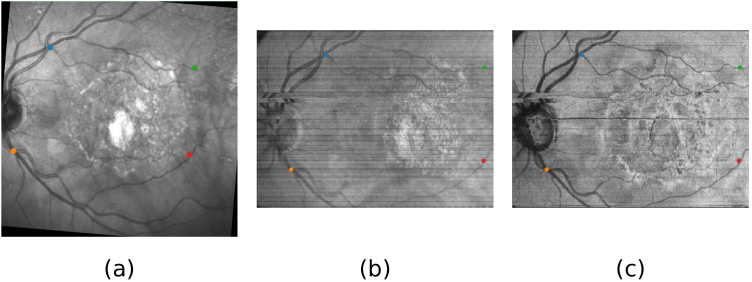
Representative example of the four corresponding keypoints (in blue, green, orange and red) for the same patient and visit on a) SLO image, b) hybrid OCT projection, and c) layer-based OCT projection.

In each pair of images in the test set, four landmark points were manually tagged (Fig. 4). These points corresponded to crossovers and bifurcations present in both the SLO and the PS-OCT intensity projections. In order to provide a representative error metric, these landmark points were spread around the field of view. To avoid any bias towards any of the different PS-OCT intensity projections previously described, the points were labeled if they were visible in any of the different projections. To obtain an error metric, we calculate the mean Euclidean distance between the four corresponding pairs of landmark points after the registration.

We then used four aggregate metrics to assess the performance of our method across the cases: (i) mean error, (ii) median error, (iii) number of registration failures, and (iv) the mean error excluding the registration failures. As a registration failure, we considered a case when the error between the corresponding keypoints was larger after the registration process than it was before it, i.e., by simply overlaying the images, without any transformation.

Additionally, to mitigate the effects of the inherent stochasticity associated with RANSAC, we conducted every experiment three times. From those, we select the result that produced the median number of registration failures.

#### 4.3.2 Z axis registration

To evaluate the Z axis registration performance, we use the outer boundary of the RPE layer as a reference. The RPE, in conjunction with the ILM, is one of the most reliable layers in terms of its detection quality. Moreover, the ILM and the RPE are two layers that are the furthest apart from one another. Therefore, this distance should be sensitive to errors in axial registration. To calculate an error metric, after registering the images, we compute the differences among the locations of the outer boundary of the RPE layers. This A-scan-wise (per-column) error is aggregated across all A-scans in different ways to produce a comprehensive set of error metrics: (i) mean error, (ii) median error, (iii) maximum mean error per B-scan, (iv) maximum mean error per A-scan, and (v) maximum error. Furthermore, also using the retinal layer segmentations, we calculate the commonly used DICE score [24,65], for the whole retinal width.

These different metrics help to evaluate the performance of the method from multiple complementary aspects. For instance, the mean error can be skewed due to the presence of outliers. However, the median error compensates for these, while the maximum error metrics can illustrate the difference of these outliers with respect to the median. It should be noted that, for the layer point matching approaches, we ran RANSAC three times per experiment to robustly report the results. Using these three runs, we provide the results for the run which obtained the mean result in terms of median error.

Due to the relative position of both 3D OCT volumes, there may not be a complete match between PS-OCT and the Spectralis data. This may cause some B-scans at the beginning or at the end of the 3D volumes to have completely missing or only partial correspondences. To address this, we only computed the error metrics in B-scans with retinal data information for both PS-OCT and Spectralis. Moreover, in the case of partial correspondences, we only computed the error metrics in the valid parts of B-scans, where both OCTs overlap. Finally, it should be noted that these error metrics are also affected by inaccuracies in the 2D registration, as they cannot be compensated by the Z-axis registration step.

## 5. Results

### 5.1 2D registration performance

**Fig. 5.**
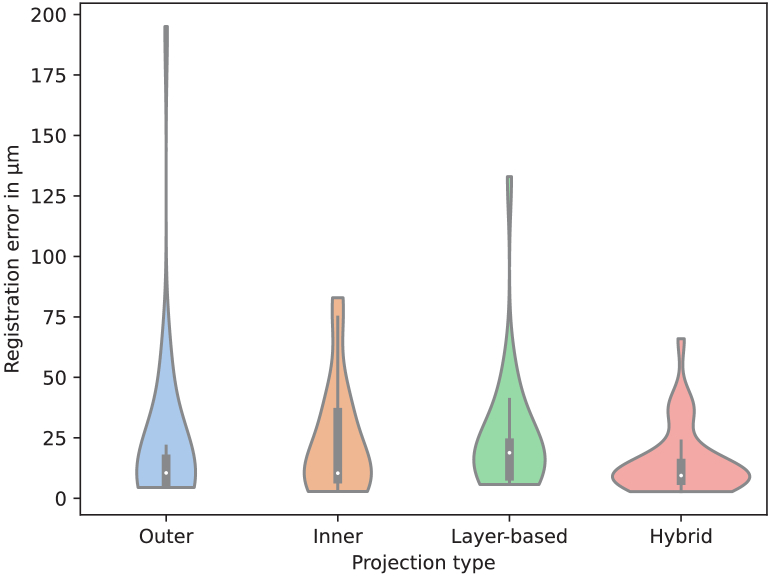
Violin plot for the results of the different projections used in the 2D registration approach, measured in µm using the average distance of the four control points in each image pair. The registration failures were removed to facilitate the comparison.

The results for the experiments regarding the PS-OCT intensity projection are shown in Table 1 and in the violin plot in Fig. 5.

**Table 1.** 2D registration results for the different OCT projections. The registration failures are measured in the number of cases in which the method fails. For both mean errors, the results also include the standard deviation across cases, also measured in µm. The best results are highlighted in bold.

Projection	# Registrations Failures	Median Error (µm)	Mean Error (µm)	Mean Error without Registration Failures (µm)
Outer	3	62.25	944.76 ± 2965.46	128.54 ± 209.5
Inner	13	247.94	10666.55 ± 22930.86	134.62 ± 133.0
Layer-based	8	124.07	2491.17 ± 9971.31	142.97 ± 151.16
Hybrid	**1**	**54.6**	**144.79 ± 326.42**	**86.96 ± 80.11**

**Fig. 6.**
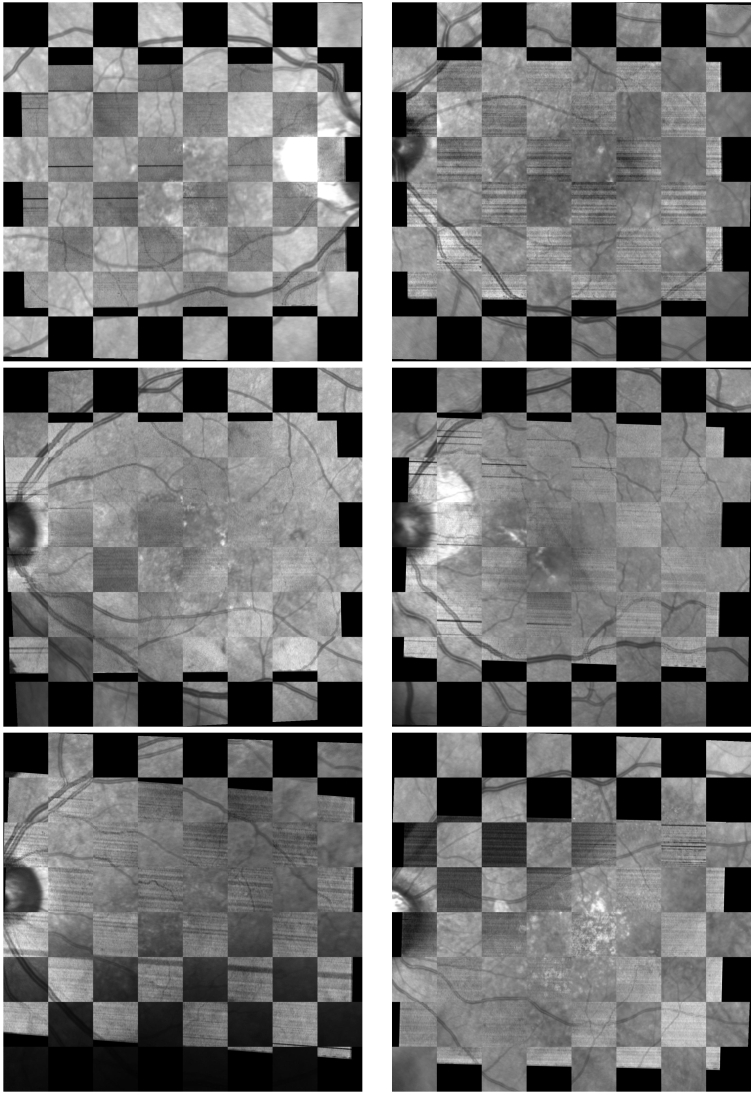
Representative en-face 2D image registration examples from the test set. Each pair of images is from a different patient. In the checkerboard pattern, the smaller image (no information on top and bottom rows, black background) is the PS-OCT intensity projection while the bigger image (the one with the first and last row with information) corresponds to the Spectralis SLO image.

The outer and hybrid projections performed better than the other two projections across all the error metrics. The hybrid projection produced the smallest median error with a statistically significant difference compared to outer projection ( p=0.045 , Wilcoxon signed rank test). Representative registration results obtained using the hybrid projection are shown in Fig. 6.

There is a clear difference in performance between the tested OCT projections. These differences are especially obvious in the number of registration failures and in the mean and median errors. The inner and layer-based projections produce more than double the median error of the hybrid projection. The high mean error of these projections, especially from the inner projection, stands out. It is produced due to the multiple degrees of freedom of the transformation, which in the case of the registration failures, can produce extremely erroneous transformations, leading to these error scores. Looking at the mean error without the registration failures, we can see that the errors are in line with the other projections. It should be noted that, the number of registration failures and the mean error without registration failures can be misleading as the cut-off point for considering whether an image is a failure depends entirely on how the images are aligned originally and this is somewhat arbitrary. In this sense, both metrics should be interpreted in conjunction with the mean and median errors.

Analyzing each of the metrics in detail for the hybrid projection, we can observe that, just by eliminating the cases considered as failures, the mean error decreases by almost half. Furthermore, this error translates to an average of just 15 pixels, where the SLO has a size of a 1536×1536 and the PS-OCT intensity projection image, scaled to be of the same pixel-spacing as the SLO image, is of size 1392×1046 . Similarly, the median error for this method in pixels is around 9 . These errors are remarkably small, especially considering the quality and size of the source images. Finally, as represented in Fig. 6 bottom row, despite having some low contrast zones, the hybrid projection still produces accurate registrations in those cases.

To complete the registration pipeline, we use the results produced by the hybrid projection in order to create the Z axis registration. It should be noted that, in the Z axis registration step, we removed the particular case where the 2D registration was deemed to fail completely.

### 5.2 Z axis registration performance

**Fig. 7.**
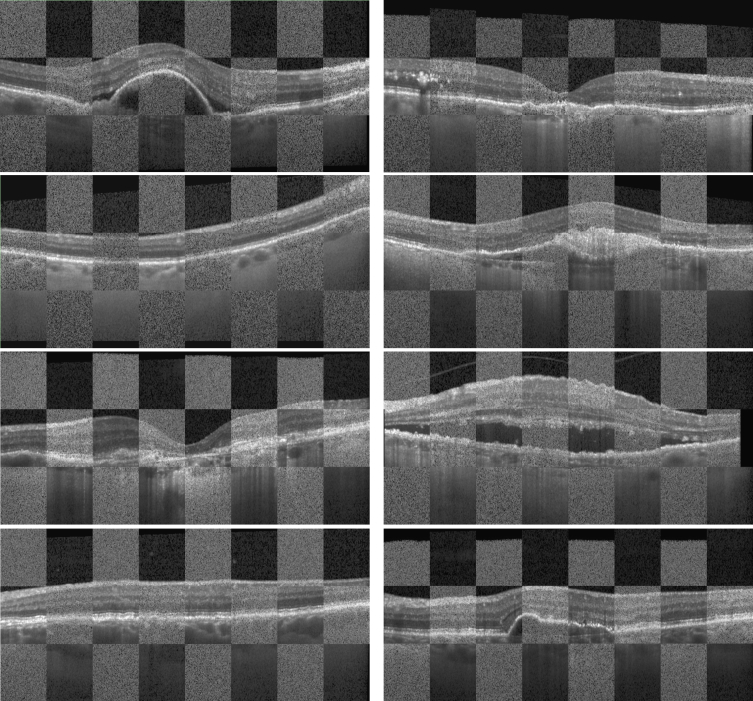
Representative Z-axis registration examples from the test set. Each pair of images is from a different patient. In the checkerboard pattern, the image with more noise corresponds to the PS-OCT and the other one to the Spectralis OCT.

The results for the Z axis registration of the PS-OCT and Spectralis images are shown in Table 2. In general, the column shifting approach outperformed the layer point matching methods across all the metrics. The median error was statistically significantly smaller ( p<0.001 , Wilcoxon signed-rank test). Representative image registrations obtained with column shifting approach using the processed layers are shown in Fig. 7. The two layer point matching methods performed similarly, indicating no significant difference between the two transformation models.

**Table 2.** Z-axis registration performance, all the metrics except DICE are measured in µm. Best results for each metric are highlighted in bold.

Model	DICE	Mean error	Median error	Max. avg. error per B-scan	Max. avg. error per A-scan	Max. error
Column Shifting	**0.98**	**12.35**	**9.33**	**27.45**	**28.83**	**162.31**
LPM w. Rigid transform	0.96	20.53	11.76	132.69	72.4	635.64
LPM w. Similarity transform	0.96	19.98	11.51	121.85	71.52	653.34

**Fig. 8.**
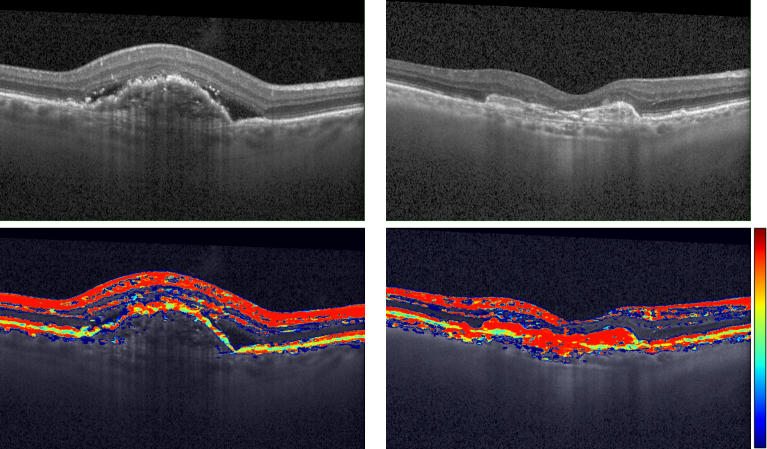
Spectralis OCT (top row) and the overlayed DOPU signal from PS-OCT mapped using the proposed registration approach (bottom row).

To illustrate how the signal from one OCT can then be mapped to the other, Fig. 8 shows an example of the DOPU signal from the PS-OCT overlayed on top of the registered Spectralis OCT in two test images.

## 6. Discussion

We presented a novel method to align 3D OCT volumes, produced by different OCT devices. We propose a two-step approach to register these volumes. First, we employ a deep learning-based 2D registration using *en-face* projections or analogous 2D representations of the volume. Second, we propose a Z-axis registration step, based on the ILM layer segmentation. Overall, the method provided accurate results in both registration steps.

For the 2D registration we used the SLO instead of the Spectralis OCT projection due to its low number of B-scans. The SLO has several advantages over the OCT projections, as it has higher image quality and higher FoV. However, it has a major drawback, as it converts the 2D registration step into a multi-modal process. Despite this, our methodology produces accurate results for this multi-modal registration. It should be noted that, in the case of other commercially available devices such as Cirrus or Topcon, their higher number of B-scans (128) would produce higher quality *en-face* projections, similar to those produced by the PS-OCT device. Therefore, we could use those projections directly, like the PS-OCT one, without modifying our methodology. That is, our proposal can work in both mono-modal and multi-modal scenarios without any change due to its training scheme. This is advantageous as it allows using the best data available depending on the type of device without any modifications.

R2D2 was shown capable of producing accurate results for this multi-modal registration. This is partly due to the high visual similarity between the two modalities. Both images (SLO and OCT projection) depict the registrable patterns, like the blood vessel tree, in a similar way. Therefore, even if the method was originally created for mono-modal registration, its contrastive training allows it to select the shared information across image modalities and thus create a cross-modal descriptor. Moreover, as the registration is between two scans acquired at the same visit, the information represented in both images contains no major deformations or changes in retinal morphology, helping the cross-modal learning process.

As previously demonstrated, R2D2 performs best when using a small reliability window [25,32]. This produces many detections, although with lower reliability than with a larger window. This puts more weight on the description and matching steps, as both must be robust and reliable to deal with the increased number of points and the increased number of matching possibilities. Therefore, reducing the random noise in the OCT projections ensures accurate description of the actual retinal features, instead of noise patterns, effectively improving the results as it allows for more consistent description. It should be noted that, although we use a standard value for k which yields accurate registrations, a detailed study of this parameter for these images could be beneficial, as different modalities have unique characteristics that may influence this parameter.

Overall, the outer and hybrid OCT projections were the ones which produced the best results across the metrics considered. The outer and the hybrid projections present low contrast but have lower amounts of noise, in comparison with the rest of the projections. On the other hand, the inner and layer-based projection produced the worst results. These two projections have a high contrast at the cost of more noise in the form of dots and lines. This leads to worse detection and description of the keypoints, which in turn leads to worse registration performance. The outer projection has less noise but also has big zones without contrast, which impedes accurate description of keypoints. Thus, the resulting registrations are accurate, but they cannot be finely adjusted in those areas, causing the bigger error metrics, when compared to the hybrid projection. This proves that, in order for the registration to be reliable, as much of the image as possible must have acceptable contrast ratios while keeping noise to a minimum.

The Z axis registration step relies on the layer segmentations, therefore, it is very important to have a high quality and reliable segmentation method. In pathological cases, large registration errors can be caused if the segmentation is inaccurate. The best approach for the Z axis registration was shown to be the column shifting. However, it should be noted that, while in the mean error the difference is notably big (more than 1.5× more), the difference in terms of median error is not as remarkable. Similarly, in terms of DICE score, while the difference between column shifting and LPM approaches is clear, it is not as notable as in the mean or peak errors of the RPE locations. This is due to the performance of the layer point matching approaches on the incomplete matches. These incomplete slices may appear at the beginning or at the end of a volume due to the relative positioning of the two volumes. These partial or incomplete matches are B-scans that do not match with another complete one. Due to the smaller number of keypoints, the layer point matching methods are less reliable as they are more likely to be fitted to noisy points, thus producing higher errors. This is clearly reflected in the difference between the mean and median error, as these outliers skew the distribution of errors and affect the mean error more than the median one. This difference is best seen in the peak errors, i.e., maximum average error per B-scan, per A-scan or maximum error, where the errors of the Layer Point Matching methods are much higher than those of the Column Shifting approach, even up to four times higher. It should be noted that we also tested the affine transformation, however, due to the higher number of degrees of freedom, these outlier errors were more exaggerated while the normal B-scan registration was not improved, so it was discarded. Finally, even if the method demonstrated to be robust against slight segmentation mistakes, big errors stemming from low-quality image capture or en-face registration represent a limitation of the Z-axis registration approach.

Our proposed method represents the first approach in the field to address multi-device OCT registration. This impedes direct comparison with previous single-device registration methods. Therefore, in order to compare our method to the rest of 3D OCT registration approaches, we would have to devise adaptations so that they could work in our experimental setting (i.e. multi-device registration). Moreover, the state-of-the-art methods are not generally available or, if they are, they have been released only in a compiled form [37] which impedes modification. Therefore, in order to compare our method with other 3D OCT registration approaches, we would have to re-implement and modify them according to our multi-device target. Finally, none of the state-of-the-art approaches published their training or testing datasets, which makes faithfully recreating and validating them even more challenging.

In terms of execution times, even though different hardware and OCT image settings impede direct comparison, state-of-the-art methods take, from hours per volume pair [36] to at least 15-30 minutes depending on the type of OCT volume, as reported in the work of Pan et al. [24]. The differences in execution times within this method [24] also highlight the complexity of comparing methods, even if disregarding the hardware settings, due to the variability among OCT devices and capture protocols.

Using a computer equipped with an AMD 5800X, 32GB of RAM and an Nvidia 3060 Ti, the 2D registration step takes around 7 minutes and 10 seconds to register each image pair, on average. From this time, around 4 seconds is spent on network inference and keypoint selection, less than 1 second is spent on the descriptor matching, around 3 minutes and 30 seconds on the RANSAC and approximately 3 minutes and 35 seconds applying the computed transformation to the moving OCT volume. Similarly, the Z-axis registration takes approximately 11 seconds using the column shifting approach, 1 minute and 15 seconds using the layer point matching method with the rigid transformation and 1 minute and 24 seconds using the layer point matching method with the similarity transformation. This way, using the chosen approach for Z-axis registration, the whole methodology would take, approximately, 7 minutes and 20 seconds per pair of OCT volumes.

The difference between the displacement approach and the layer point matching is due to the use of RANSAC. Similarly, in the 2D registration a significant portion of the time is spent in RANSAC as well. This is due to the stochastic nature of RANSAC as we provide it with a high budget in order to limit its inherent randomness. This makes the results more stable and thus more robust to RANSAC’s randomness at the cost of computational time. RANSAC’s computational complexity can be described as $O(T_{iter}(C_{est}(m)+NC_{fit}))$ [66], where $NC_{fit}$ is the cost to compute how each element fits the model, $C_{est}(m)$ is the estimation cost of a model m and $T_{iter}$ is the number of iterations. Therefore, even if the cost of fitting each element and of estimating our model is relatively low (we use affine, similarity or rigid transformations, depending on the step) the repetition due to the multiple iterations increases the execution time. This is exemplified by the difference in computation time between the layer point matching with rigid and with similarity transformations. In this case, every parameter is the same except the cost of fitting the model. This results in approximately 10 extra seconds of processing due to the increase in complexity derived from using the similarity transform.

Overall, our method obtains fast execution times in the target task of multi-device OCT registration. Although the obtained times can not be fairly compared to other approaches due to differences in hardware and in the OCT volumes themselves, we can conclude that our method produces execution times comparable to current state-of-the-art methods.

## 7. Conclusions

In this paper we propose a novel two-step registration method to align 3D OCT volumes, specifically those produced by different OCT devices. This is the first method to address the task of multi-device OCT registration in the state of the art. The first step of our proposal is a feature-based deep learning 2D registration, which is able to register the OCT volumes *en-face*. This registration is casted as a multi-modal registration between 2D representations of the volume (SLO images and OCT *en-face* projections). The second step is Z-axis registration based on the retinal layer segmentation, particularly the ILM. The proposed method obtains accurate results and fast execution times in our experiments.

The proposed method provided accurate results in both registration steps and in a 3D evaluation. Our approach has several advantages like using unsupervised deep learning for the 2D registration as it does not need a ground truth to learn and can be easily adapted to other 2D representations of the OCT volumes simply by retraining the network. Moreover, the Z-axis registration can work with any retinal surface and any segmentation method. However, limitations can arise from an incorrect 2D registration, in which case, the Z-axis registration would not provide accurate results. Nevertheless, the method’s modular nature allows for independent modifications on the two registration steps. Potential future improvements include introducing image pre-processing in the 2D registration to highlight key structures, creating a hybrid approach for the Z-axis registration which combines column shifting and layer point matching and incorporating information from neighboring B-scans to include contextual data.

Registering images from different OCT devices results in images that combine their best features. This can allow to map certain features from one device to another that does not produce them, like the DOPU signal in the case of PS-OCT or the angiography signal from an OCTA device. In particular, in our experimental setting, we demonstrate the mapping of the DOPU signal from a PS-OCT image to the Spectralis OCT. This can help in the layer segmentation process of the Spectralis OCTs due to the tissue specific contrast provided by PS-OCT. Moreover, registering PS-OCT and conventional OCT, can allow to train deep neural networks and use them to retrospectively extract the extra information provided by the polarized light source, e.g., DOPU signal, from already collected scans as demonstrated in [9]. In future work we will test the proposed method using other devices like the ones provided by Zeiss and Topcon or different modalities such as OCTA.

## Data Availability

Data underlying the results presented in this paper are not publicly available at this time but may be obtained from the authors upon reasonable request.
